# Multiplexed electrochemical liposomes applied to the detection of nucleic acids for Influenza A, Influenza B and SARS-CoV-2

**DOI:** 10.1007/s00216-024-05145-8

**Published:** 2024-01-19

**Authors:** Florian Gerstl, Michael Loessl, Veronika Borggraefe, Antje J. Baeumner

**Affiliations:** Institute of Analytical Chemistry, Chemo- and Biosensors, University of Regensburg, Universitätsstraße 31, 93053 Regensburg, Germany

**Keywords:** Multiplex, Electrochemical biosensor, Laser-induced graphene, DNA, Liposomes, SARS-CoV-2

## Abstract

Multiplexing is a relevant strategy for biosensors to improve accuracy and decision-making due to the increased amount of simultaneously obtained information. Liposomes offer unique benefits for label-based multiplexing since a variety of different marker molecules can be encapsulated, leading to intrinsic signal amplification and enabling a variety of detection formats. We successfully developed an electrochemical (EC) liposome-based platform technology for the simultaneous detection of at least three analytes by studying parameters to ensure specific and sensitive bioassay performance. Influenza A and B and SARS-CoV-2 sequences served as model system in a standard sandwich hybridization assay. Studies included encapsulants, probe distribution on liposomes and capture beads, assay setup and interferences between liposomes to also ensure a generalization of the platform. Ruthenium hexamine(III), potassium hexacyanoferrate(II) and *m*-carboxy luminol, when encapsulated separately into a liposome, provided desirable long-term stability of at least 12 months and no cross-signals between liposomes. Through the optimization process, low limits of detections of 1.6 nmol L^−1^, 125 pmol L^−1^ and 130 pmol L^−1^, respectively, were achieved in a multiplexed assay setup, which were similar to singleplex assays. Non-specific interactions were limited to 25.1%, 7.6% and 7.5%, respectively, through sequential liposome incubations and singleplex capture bead designs. Here, ruthenium hexamine liposomes had only mediocre performance so that low overall signal strength translated into higher LODs and worse specificity. A different marker such as ferroin may be an option in the future. The identification of further electrochemical markers will provide new opportunities for liposomes to function as multiplex, orthogonal or internal standard labels in electrochemical bioassays.

**Graphical Abstract:**

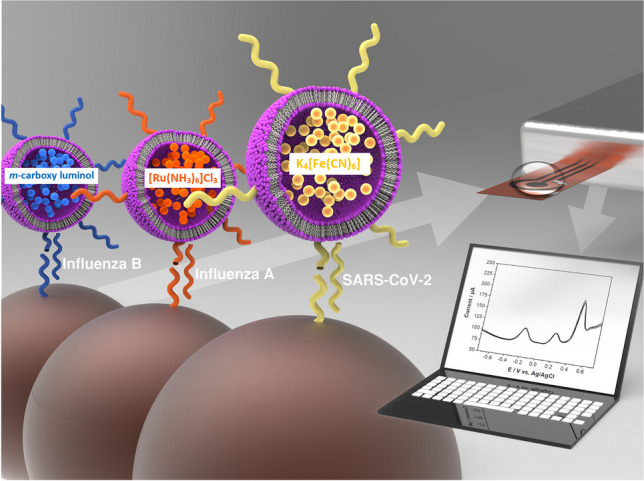

**Supplementary Information:**

The online version contains supplementary material available at 10.1007/s00216-024-05145-8.

## Introduction

Biomarkers play important roles in disease diagnosis and treatment [1, 2]. However, diagnosis based on a single biomarker can be inaccurate and some diseases even require precise diagnosis of several biomarkers to be appropriately identified [1–3]. To avoid misdiagnosis with potentially severe consequences for the patient, biosensors with low limits of detections and the ability to detect several analytes simultaneously become increasingly relevant for environmental and healthcare monitoring [1–3]. Ideally, the simplicity of a singleplex assay is maintained in the multiplex format while the amount of information per test increases dramatically [1, 3].

The two major strategies achieving multiplexing are (i) spatial separation of transducer elements modified with individual biorecognition elements resulting in array like structures or (ii) the use of multiplex labels for each analyte, which allow simultaneous detection [1]. Label-based multiplexing has the risk of cross talk between overlapping labels, whereas spatial multiplexing results in a more complex and expensive sensor fabrication and integration [2], which is often undesired for single-use point-of-care tests.

Unfortunately, most labels only have a 1:1 signalling ratio between analyte and label, resulting in lower sensitivities. Enzymes, some functional nanomaterials, and liposomes have an intrinsic signal amplification, where a single binding event leads to a signal increase of orders of magnitude [4–6].

Liposomes are artificial, spherical nanovesicles with a lipid bilayer encapsulating an aqueous cavity. This allows them to serve as carriers for large amounts of both hydrophilic and hydrophobic molecules by encapsulating them in the inner cavity or into the lipid bilayer, respectively [3, 6, 7]. The surface of the lipid bilayer can be modified to induce functionality, resulting in many academic and commercial applications in drug and gene delivery, vaccination and biosensing [6, 7]. The combination of generic signal amplification and the ability to easily encapsulate a variety of different molecules makes liposomes ideal candidates for label-based multiplexing.

In multiplex setups, liposomes themselves can serve as labels as demonstrated by Wogelred *et al.* and Gunnarsson *et al.*, who used liposomes as chemical barcodes for time-of-flight secondary ion mass spectrometry for the detection of GM1 and biotin or amyloidβ and Tau [8] or two target DNAs [9]. However, more commonly liposomes only serve as the carrier for the actual multiplex label. Beloglazova *et al*. reported an immunoassay with silica-coated liposomes loaded with quantum dots for the detection of mycotoxins zearalenone and aflatoxin B1 in cereals [10]. Zhou *et al.* used liposome-quantum dot complexes for the duplex detection of single-stranded DNA [11]. Chaize *et al.* used fluorescent liposomes encapsulating Cy5 or modified with lipid grafted rhodamine to confirm their localized binding to complementary oligonucleotides [12]. Johari-Ahar *et al.* encapsulated Cd^2+^ and Cu^2+^ into liposomes for the electrochemical detection of the two cancer biomarkers epidermal growth factor receptor (EGFR) and vascular endothelial growth factor (VEGF) [13]. Zhong *et al.* have reported a duplex immunoassay for neuron-specific enolase and pro-gastrin-releasing peptide using electrochemical liposomes encapsulating uric acid and ascorbic acid [3]. In fact, multiplexing using electrochemical liposomes is utterly underreported considering the great attention of liposomes for electrochemical biosensors [6]. The principle of location-separated electrochemical detection with liposomes was already shown by Wongkaew *et al.* [14]. Still, electrochemical sensors are often preferred for home-testing and point-of-care applications, due to their high accuracy, good sensitivities, rapid and easy detection, cost-effectiveness and simple instrumentation and miniaturization [1, 2, 6]. Combining these advantages with the increased amount of obtained information through multiplexing is currently challenging but will offer significant benefits as multiparameter analyses can increase the confidence of data interpretation, or multiple common analytes can be determined for cost and time reduction. Therefore, research on electrochemical multiplexing is highly relevant.

To further investigate the possibility for electrochemical multiplex sensors involving liposome labels, we developed an electrochemical platform for the simultaneous detection of three analytes using a nucleic acid hybridization assay for DNA sequences derived from NASBA amplicons of Influenza A, Influenza B and SARS-CoV-2 as model analytes. The three EC markers selected were ruthenium hexamine(III), potassium hexacyanoferrate(II) and *m*-carboxy luminol each encapsulated separately into a liposome and tagged with a specific reporter probe. The three EC-liposomes can easily be supplemented by further markers in the future and are thus a simple and powerful strategy for multiplexed biosensing.

## Materials and methods

### Chemicals and materials

3,4-Dihydroxy-9,10-dioxo-9,10-dihydroanthracene-2-sulfonic acid (Alizarin Red S), cholesterol, Cresyl violet acetate, disodium ethylenediaminetetraacetic acid (EDTA), ferroin solution (25 mmol L^−1^), hexaamineruthenium(III) chloride, 1,1′-dimethyl[4,4′-bipyridine]-1,1′-diium dichloride (methyl viologen), NADH disodium salt, 4-nitrophenol, potassium hexacyanoferrate(II) tetrahydrate, resazurin, Sephadex G-50 medium, sodium citrate dihydrate, TWEEN 20 and uric acid were purchased from Sigma-Aldrich. Bovine serum albumin fraction V (BSA), disodium hydrogen phosphate dihydrate, formamide, glycine, hydrochloric acid, methylene blue, neutral red, potassium dihydrogen phosphate and sodium azide were purchased from Merck. Ascorbic acid, calcium dichloride, d(+)-sucrose, ficoll 400, n-Octyl-β-d-glucopyranoside (OG), sodium chloride and sodium hydroxide were purchased from Carl Roth. 1,2-Dipalmitoyl-sn-glycero-3-phosphocholine (DPPC), 1,2-dipalmitoyl-sn-glycero-3-phospho-(1′-rac-glycerol) sodium salt (DPPG) and the extrusion kit and membranes were purchased from Avanti Polar Lipids. Chloroform, 2-[4-(2-hydroxyethyl)piperazin-1-yl]ethanesulfonic acid (HEPES) and methanol were purchased from VWR. 2-Amino-2-(hydroxymethyl)propane-1,3-diol (Tris) was purchased from affymetrix (Cleveland, OH, USA). *m*-Carboxy luminol was synthesized by TAROS Chemicals (Dortmund, Germany). All buffers and aqueous solutions were prepared with Milli-Q water (≥ 18.2 MΩ cm, Merck).

Kapton 500 HN was produced by DuPont. Conductive silver ink (item 530042) was purchased from DODUCO Contracts and Refining (Pforzheim, Germany). Streptavidin-coated paramagnetic beads (Dynabeads MyOne Streptavidin C1) were purchased from Invitrogen/Thermo Fisher Scientific. 3′-Cholesterol-TEG-modified reporter probes were purchased from biomers.net (Ulm, Germany). Target oligos and 5′-biotin-modified capture probes were purchased from metabion (Planegg/Steinkirchen, Germany).

### Design of multiplex DNA probes and targets

First NASBA amplicons of Influenza A, Influenza B and SARS-CoV-2 were identified by applying the multiplex NASBA probes from Xing *et al*. [15] to BLAST searches. The obtained amplicon sequences from accessions (Influenza A MP1: MW855514.1; Influenza B MP1 MT314580.1; SARS-CoV-2 (S gene): OK335513.1) were then considered as targets. DNA probes against these targets for the sandwich assay were designed using UniqueProbeSelector 2.0 [16]. The obtained 40-nt-long probes were split in half for a capture and a reporter probe. The corresponding sequences were used as target oligos in this work. Several different sets of probes and targets were compared and optimized using Thermo Fisher Multiple Primer Analyzer, mfold [17] and PrimerDimer [18] to obtain similar physical properties and the least number of potential cross-hybridizations between all six probes and three targets. Probe specificity was confirmed by another BLAST search. The chosen sequences are show in Table 1.

**Table 1:** Probe and target oligonucleotides

Name	Sequence with modifications (5′→3′)	Bases
Inf A capture probe	[biotin]-CAAGATCTGTGTTCTTTCCT	20
Inf A target	AGACTGGAAAGTGTCTTTGCAGGAAAGAACACAGATCTTG	40
Inf A reporter probe	GCAAAGACACTTTCCAGTCT-[TEG-cholesterol]	20
Inf B capture probe	[biotin]-CAGAGAGTACTTCCTTCATTG	21
Inf B target	GAAATCCAGGCCAAAGAAACAATGAAGGAAGTACTCTCTG	40
Inf B reporter probe	TTTCTTTGGCCTGGATTTC-[TEG-cholesterol]	19
SC2 capture probe	[biotin]-TTATCAGGGTGTTAACTGCAC	21
SC2 target	GAATAGCAACAGGGACTTCTGTGCAGTTAACACCCTGATAA	41
SC2 reporter probe	AGAAGTCCCTGTTGCTATTC-[TEG-cholesterol]	20

### Liposome preparation

Liposomes were prepared using reverse-phase evaporation according to the protocol from Edwards *et al.* [19] modified for different encapsulants [20, 21]. 30 mg (40.9 µmol) DPPC, 15 mg (20.1 µmol) DPPG and 19 mg (49.1 µmol) cholesterol were dissolved in a mixture of 3 mL chloroform and 0.5 methanol using short sonication. 37.5 µL of 400 µmol L^−1^ reporter probe (15 nmol) and 2 mL encapsulant solution (100 mmol L^−1^ Ru(NH_3_)_6_Cl_3_, 150 mmol L^−1^ NaCl in 20 mmol L^−1^ HEPES pH 7.5 or 200 mmol L^−1^ K_4_[Fe(CN)_6_] in 20 mmol L^−1^ HEPES pH 7.5 or 75 mmol L^−1^ *m*-carboxy luminol in 200 mmol L^−1^ HEPES pH 7.5 with additional 20% (v/v) 1 mol L^−1^ NaOH) were added. The organic solvents were removed by rotary evaporation (55 °C water bath; 10 min at 600 mbar, 10 min at 500 mbar and 20 min at 400 mbar). Then, another 2 mL of encapsulant solution was added. The suspension was vortexed for 5 min with short reheating steps in between to keep the temperature above the phase transition temperature. Followed by a second rotary evaporation step (55 °C water bath; 20 min at 380 mbar and 20 min at 280 mbar). Liposomes were extruded 21 times through polycarbonate membranes with pore sizes of 0.4 µm and 0.2 µm and gel filtered on a Sephadex G-50 medium column (length 9 cm) using HSS buffer (for FCN and RuHex: 10 mmol L^−1^ HEPES, 200 mmol L^−1^ NaCl, 250 mmol L^−1^ sucrose, 0.01% NaN_3_, pH 7.5) or Glycine-NaOH buffer (for mCL: 10 mmol L^−1^ glycine, 200 mmol L^−1^ NaCl, 225 mmol L^−1^ sucrose, 0.01% NaN_3_, pH 8.5) as eluent. Liposomes were dialyzed against 1 L of their respective eluent buffer for 16 h using dialysis membranes with a molecular cutoff value of 12–14 kDa (SpectrumLabs, Standard RC tubing). Liposomes were stored at 4 °C in the fridge for more than 12 months.

### Liposome characterization

A Malvern Zetasizer Nano ZS (Malvern Instruments) was used to determine liposome size distributions by dynamic light scattering (DLS) and zeta potentials by zeta potential measurements. Liposomes were diluted 1:100 in HSS buffer for these experiments.

ICP-OES with a SpectroBlue FMX36 TI/EOP (SPECTRO Analytical Instruments) was used to determine the phosphorus content and thus the phospholipid concentration. Liposomes were diluted 1:150 in 0.5 mol L^−1^ HNO_3_. The total lipid concentration was then calculated from the phospholipid concentration.

Encapsulant concentrations were determined by comparing lysed and non-lysed liposomes with calibration curves of the respective encapsulant with/without surfactant.

### Laser-induced graphene

Laser-induced graphene (LIG) was produced under ambient conditions by laser irradiation of Kapton HN 500 polymide foil with a VLS 2.30 laser engraving system (Universal Laser Systems, 30 W CO_2_ laser, *λ* = 10.6 µm). Laser settings had been optimized by Behrent *et al.* previously [22]. A 2″ lens was used in combination with 1% power (0.3 W), 10% speed (12.7 cm s^−1^), 1000 PPI and image density 6.

### One-step sandwich hybridization assay

The one-step assay was similar to previous work [20]. Microwell plates (greiner bio-one 655101) were washed once with 120 µL washing and blocking buffer (0.05% (v/v) TWEEN 20, 0.01% BSA in PBS pH 7.4) and once with 120 µL hybridization buffer (HB) (1.35 mol L^−1^ NaCl, 0.135 mol L^−1^ sodium citrate, 30% formamide, 0.2% ficoll 400, 0.01% NaN_3_, pH 7.0). Magnetic beads (MBs) (Dynabeads MyOne Streptavidin C1) (5 µL per sample) were washed twice with 10 times the original volume of binding and washing buffer (B&W buffer) (0.5 mmol L^−1^ EDTA, 1 mol L^−1^ NaCl in 5 mmol L^−1^ Tris-HCl pH 7.5) and once with 10 times the original volume of HB. The MBs were resuspended in their original volume in HB. MBs (5 µL), capture probes (1.25 pmol), reporter probe-modified liposomes (500 µmol L^−1^ total lipid) and varying amounts of target oligonucleotides were mixed in a total volume of 100 µL and incubated for 30 min at RT while shaken at 500 rpm. Afterwards, the MBs were separated and washed three times with 120 µL HB. The liposomes bound to MBs were lysed with 30 µL of lysis solution (30 mmol L^−1^ OG in PBS) for 15 min. Finally, the lysed solution was transferred on LIG electrodes and measured with square wave voltammetry (SWV).

### Multi-step sandwich hybridization assay

Microwell plates were washed once with 120 µL washing and blocking buffer and once with 120 µL HB. MBs (15 µL per sample) were washed three times with 10 times the original volume of B&W buffer and then resuspended in their original volume in B&W buffer. The MBs were split into three equal portions and incubated for 30 min with one of the three capture probes. MBs were washed twice with 10 times the volume of B&W buffer and once with 10 times the volume of HB. 5 µL of every MB-CP combination was mixed with target oligonucleotides in a total volume of 100 µL in HB and incubated for 30 min at 500 rpm, followed by three washing steps with 120 µL HB. Then, the MBs were subsequently incubated for 20 min with each of the three liposomes (500 µmol L^−1^ total lipid), always followed by three washing steps with 120 µL HB in between. After the final washing step, 30 µL of lysis solution was added and incubated for 15 min. Finally, the lysed solution was transferred onto LIG electrodes and measured with SWV. The assay principle is illustrated in Schematic 1.

**Schematic 1:**
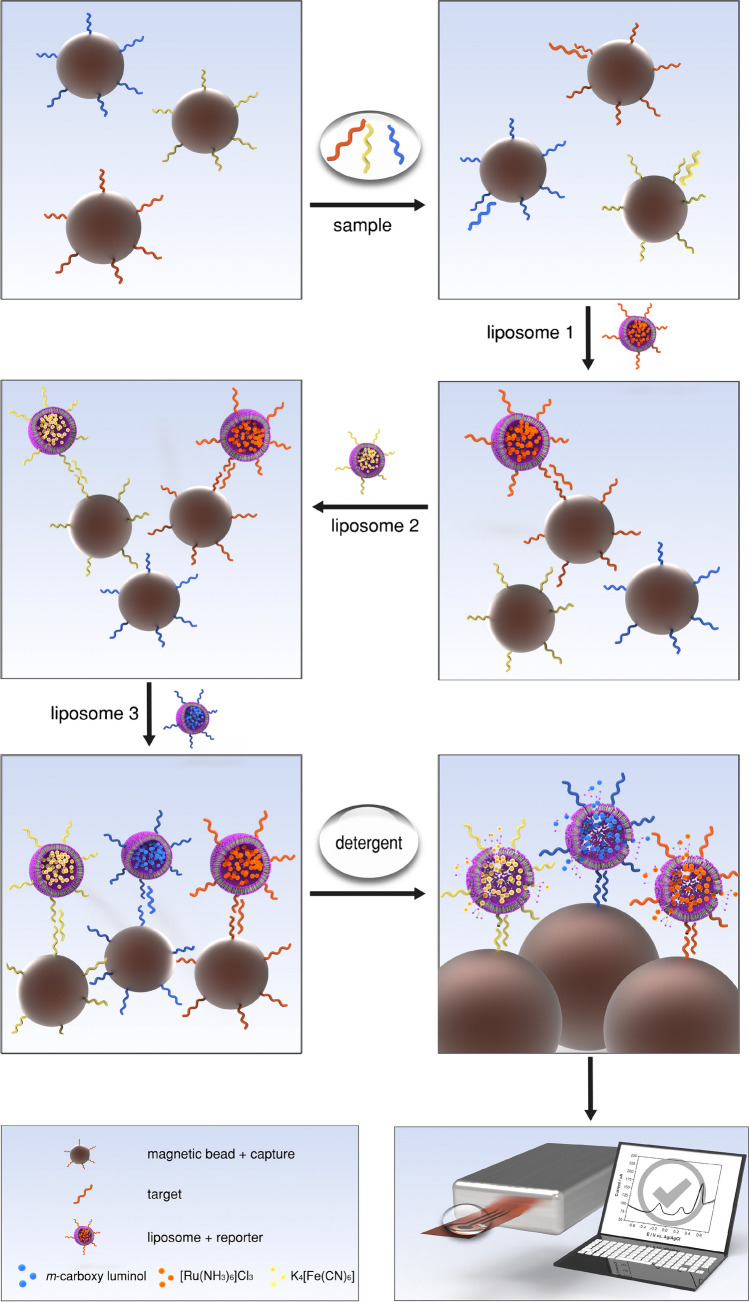
Multi-step, multiplex assay principle. Magnetic beads with immobilized capture probes (1) are incubated with a sample solution containing potential target DNAs. Afterwards, magnetic beads with hybridized targets (2) are subsequently incubated with all three liposomes modified with respective reporter probes for 20 min each (3–5). After each incubation step, magnetic separation and washing steps are performed. Finally, a detergent is added to lyse liposomes bound to the surface of magnetic beads (6). The lysate is analysed by square wave voltammetry on LIG electrodes (7)

### Electrochemical measurements

Electrochemical measurements were performed on LIG electrodes with a pseudo-Ag/AgCl reference electrode made from silver ink (Fig. S1). Electrodes were 1 day old for regular experiments and 5 days for the encapsulant stability study. Square wave voltammetry measurements were performed with a PalmSens 4 potentiostat (PalmSens BV, The Netherlands) controlled by the PSTrace 5.9 software. Samples were pretreated at −0.7 V for 3 s, followed by a scan from −0.7 V to +0.7 V with 5 mV step potential, 50 mV amplitude and a frequency of 5 Hz.

### Data analysis

All data evaluation was performed with Excel (Microsoft Corporation) and plotted in Origin 2019 (OriginLab). Data is given as mean ± standard deviation. Limits of detections were determined by ${y}_{blank}+3\times {\sigma }_{blank}$.

## Results and discussion

Liposomes can easily be synthesized with a variety of different encapsulants, hence offering various detection strategies. This can be used for the development of multiplexed bioassays where each encapsulant represents a different analyte. Here, we focused on the development of a liposome-based multiplexed electrochemical platform applied to the detection of three different DNA sequences derived from viruses.

### Liposome encapsulants for multiplexing

**Fig. 1:**
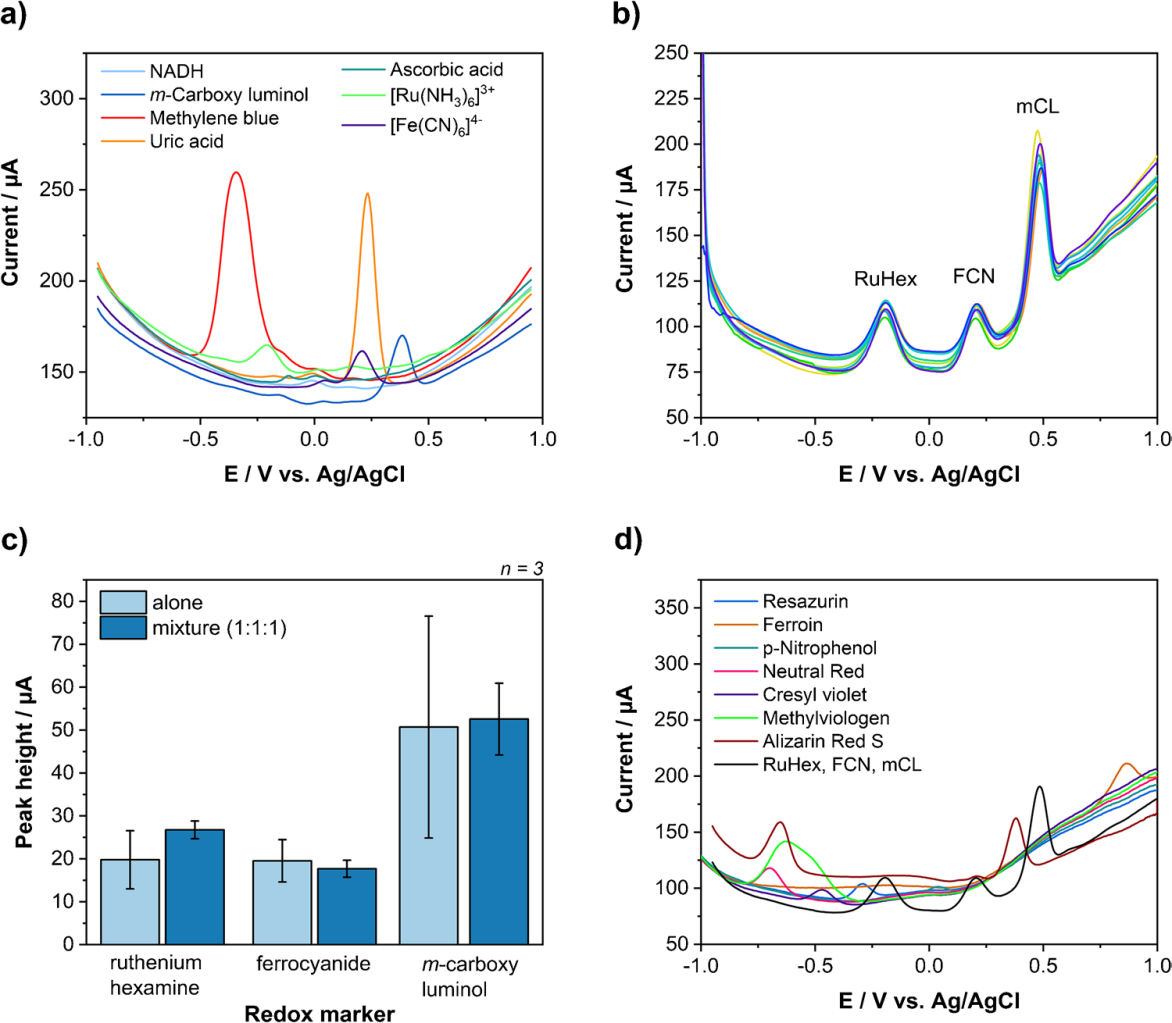
(**a**) Square wave voltammograms of NADH, mCL, methylene blue, uric acid, ascorbic acid, RuHex and FCN at 100 µmol L^−1^. Scanned from −1 V to +1 V. (**b**) Square wave voltammogram of mixtures containing 100 µmol L^−1^ [Ru(NH_3_)_6_]Cl_3_, K_4_[Fe(CN)_6_] and m-carboxy luminol. (**c**) Comparison between signal heights of 100 µmol L^−1^ [Ru(NH_3_)_6_]Cl_3_, K_4_[Fe(CN)_6_] and m-carboxy luminol alone and in mixture. (**d**) Square wave voltammograms of resazurin, ferroin, p-nitrophenol, neutral red, cresyl violet, methyl viologen and alizarin red S as potential encapsulant additions and a mixture of RuHex, FCN and mCL for comparison

The identification of suitable encapsulants is the most important aspect for a label-based multiplex approach using liposomes. Square wave voltammetry was chosen as detection strategy due to its superior performance in comparison to other voltametric approaches in previous studies [22]. Hence, the main encapsulant selection criteria were peak separation, signal performance and water solubility. Other less important criteria were toxicity, cost and commercial availability. First, square wave voltammograms were recorded for each compound individually using a concentration of 100 µmol L^−1^ (Fig. 1a). Then, potential combinations were identified and tested. Methylene blue and uric acid stood out because of their strong peak heights, which may be due to an up-concentration at the laser-induced graphene electrode via π-stacking similar to other aromatic compounds [23]. The only possible combination with at least three encapsulants had to involve *m*-carboxy luminol (mCL) due to its uniquely placed peak at 0.45 V (vs. Ag/AgCl). Unfortunately, this would lead to a poor peak separation with uric acid. Furthermore, the low water solubility of uric acid would negatively affect the encapsulation efficiency during liposome preparation. Therefore, potassium hexacyanoferrate(II) (FCN) was chosen over uric acid. Testing mixtures of methylene blue, FCN and mCL showed inconsistent results and it turned out that methylene blue was reduced by both FCN and mCL (Fig. S2). Methylene blue was hence substituted by ruthenium hexamine(III) (RuHex). This mixture showed good peak separation (Fig. 1b) and similar performances when alone or in mixture (Fig. 1c) and was therefore chosen as liposome encapsulants, albeit RuHex and FCN consistently showed lower signal intensities than mCL. The high variation in the mCL signal in this particular study was likely caused by different hand-made electrodes. Later studies demonstrated again normal standard deviations supporting this interpretation.

Additional possible encapsulants were searched to increase the multiplexing capabilities in the future and go beyond a triplex assay. Mainly chemicals with redox potentials more negative than RuHex or more positive than mCL were studied to avoid overlaps with the existing encapsulants (Fig. 1d). However, it was found that one or two of the originally selected markers would need to be dropped for this purpose. Specifically, Alizarin Red S (−0.65 V, +0.4 V) overlapped with mCL. Methyl viologen (−0.6 V) is toxic and the broad peak makes evaluation at lower concentrations very difficult. Neutral red (−0.7 V) is membrane permeable and can cause genetic defects, which suggests that it may not encapsulate sustainably in liposomes. 4-Nitrophenol (+0.05 V) overlapped with FCN. Ferroin (+0.85 V, −0.2 V) could only be used as a replacement for RuHex, because of the additional signal around −0.2 V. Cresyl violet (−0.45 V) cross-reacted in mixtures similar to methylene blue (Fig. S3). Thus, multiplexing using SWV and liposomes beyond a triplet is feasible but will require other marker combinations than those including RuHex, FCN and mCL. Anthraquinone, thionine, ferrocene carboxylic acid and Co(bpy)^3+^[24] and heavy metal ions like Cd^2+^, Cu^2+^, Pb^2+^ or Zn^2+^ [13, 25, 26] have been used by other researchers for up to quadruplex electrochemical detections and can be investigated as encapsulants for liposomes to open up new combinations for quadruplex or pentaplex strategies in the future. Here, a triplex assay was developed using RuHex, FCN and mCL as EC liposome encapsulants.

### Liposome characterization and stability study

Liposomes were synthesized using standard protocols and characterized by their hydrodynamic diameter, polydispersity index (PdI), zeta potential, final lipid and encapsulant concentrations. The initial characterizations after synthesis for all liposome batches are summarized in Table 2, which shows negligible batch-to-batch variations for the same encapsulant.

**Table 2:** Summary of liposome characterizations

Encapsulant/reporter probe	*z*-average diameter (nm)	Zeta potential (mV)	PdI	Total lipid concentration (mmol L^−1^)	Encapsulant total (µmol L^−1^*)	Encapsulant outside (µmol L^−1^*)
RuHex/Inf B	187 ± 2	−21 ± 2	0.09 ± 0.04	17.9 ± 0.1	180 ± 20	n. a.
RuHex/Inf A	184 ± 2	−21 ± 2	0.05 ± 0.02	17.2 ± 0.1	173 ± 11	n. a.
FCN/Inf A	157 ± 3	−19 ± 2	0.05 ± 0.02	16.41 ± 0.09	283 ± 9	19 ± 3
FCN/SC2	159 ± 4	−22 ± 1	0.06 ± 0.03	17.97 ± 0.09	248 ± 6	23 ± 4
mCL/SC2	145 ± 3	−9 ± 2	0.094 ± 0.004	17.93 ± 0.08	132	3 ± 2
mCL/Inf B	141 ± 2	−18 ± 1	0.08 ± 0.03	17.0 ± 0.3	128	11 ± 2

*µmol L^−1^ per mmol L^−1^ total lipid

**Fig. 2:**
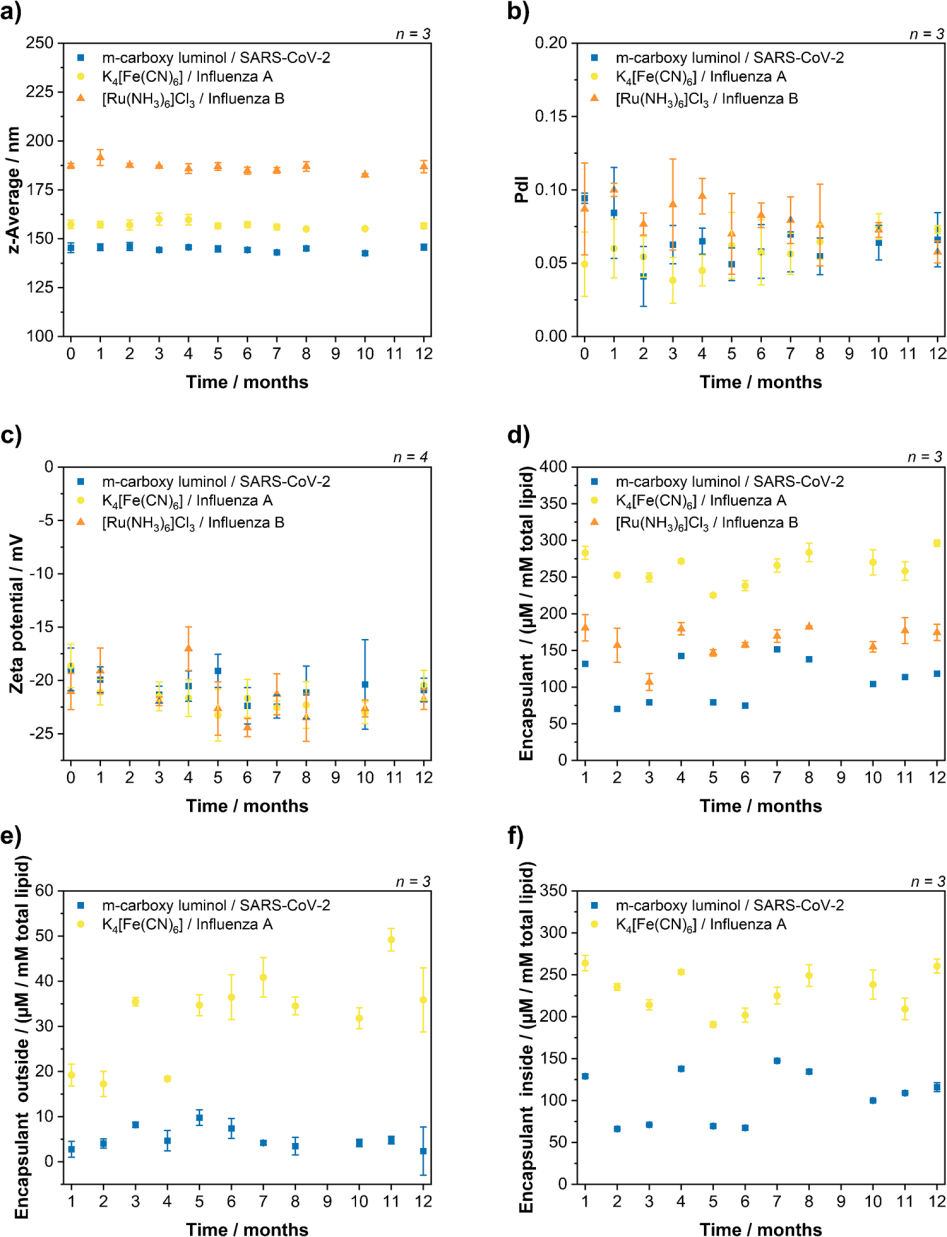
Long-term stability study of liposomes. RuHex/InfB, FCN/InfA and mCL/SC2 liposomes were characterized and monitored over 12 months. The *z*-average of the hydrodynamic diameter (**a**) and the PdI (**b**) were determined by DLS measurements in HSS buffer. The zeta potential (**c**) was determined by zeta potential measurements in the HSS buffer. The total encapsulant concentrations (**d**) and the encapsulant concentration outside of liposomes (**e**) were measured on LIG electrodes in PBS buffer and compared with respective calibration curves. The encapsulant concentration inside (**f**) was then calculated from these obtained values

They were monitored for storage over a period of 12 months (Fig. 2) indicating excellent stability with respect to size, PdI and zeta potentials. RuHex liposomes had a *z*-average of 187 ± 2 nm, FCN liposomes 157 ± 3 nm and mCl liposomes 145 ± 3 nm. The difference in size is typically observed for different encapsulants and caused by different interface activities. The PdIs were below 0.1 indicating a good, low size distribution of the liposomes, and the average zeta potentials with −21.5 mV (RuHex), −21.6 mV (FCN) and −20.7 mV (mCL) indicate good colloidal stability. Furthermore, the total encapsulant concentrations remained the same over 12 months indicating no chemical degradation of the encapsulants. Small deviations between months were a result of batch-to-batch variations of the LIG electrodes used in the analysis. Considering the variation in detection between the months due to differently made electrodes, no or minimal signal variation is observed for FCN and mCL indicating the stability of these liposomes over time, which was expected when compared to similar liposomes published earlier [21, 27]. Determining the concentration of leaked RuHex was not possible within the setup, because in samples without surfactant the RuHex signal overlapped with one derived by solubilized oxygen (−0.4 V), which presumably was a result of air entrapment in the porous LIG surface. OG used for liposome lysis to determine the total encapsulant concentration also reduced the surface tension of the solution and therefore prevented the air entrapment.

### Development of a multiplex liposome strategy

**Fig. 3:**
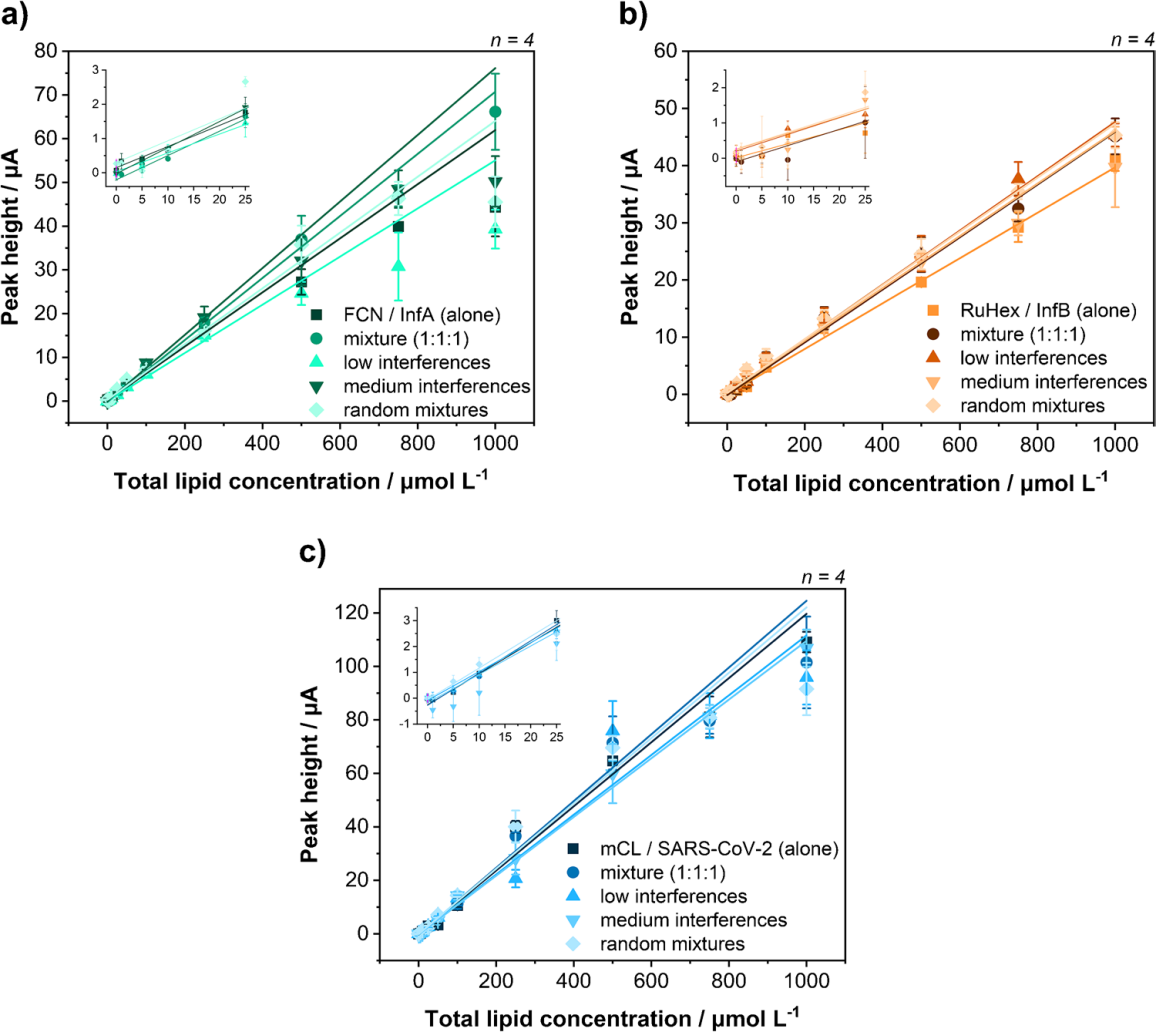
Systematic cross-reactivity study of multiplex electrochemical liposomes. Dose-response curves of lysed FCN/InfA (**a**), RuHex/InfB (**b**) and mCL/SC2 (**c**) liposomes measured in PBS with 30 mmol L^−1^ OG on LIG electrodes. Liposomes were recorded individually, in mixtures at equal concentrations, in mixtures with fixed concentrations of the other two liposomes and random mixtures. The fixed interferant concentrations were 25 µmol L^−1^ of FCN/InfA and mCL/SC2 or 50 µmol L^−1^ of RuHex/InfB liposomes (low interferences) or 250 µmol L^−1^ (medium interferences)

Possible interferences between liposomes were systematically investigated through a series of experiments with different liposome mixtures. Liposomes alone were compared to mixtures with identical concentrations of all three liposomes, mixtures with fixed low or medium concentrations of the other liposomes and random mixtures (Fig. 3). The low concentrations chosen for the interfering liposomes were 25 µmol L^−1^ for FCN/InfA and mCL/SC2 liposomes and 50 µmol L^−1^ for RuHex/InfB liposomes, due to their lower sensitivity. The medium concentrations for interfering liposomes were 250 µmol L^−1^ for all liposomes. Under all conditions, no systematic deviations were found, and it can be concluded that no cross reaction between the chosen encapsulants and liposomes is observed. The limits of detections on LIG for single liposomes were 4–7 µmol L^−1^ total lipid for mCL/SC2 liposomes, 10 µmol L^−1^ for FCN/InfA liposomes and 9–17 µmol L^−1^ for RuHex/InfB liposomes.

Target DNA sequences were detected using a sandwich approach, in which liposomes tagged with a reporter probe, target and superparamagnetic beads coupled to capture probes were mixed, isolated through a magnet, washed, lysed and subsequently added on a LIG electrode for detection (Fig. S4). The results were as expected considering previous experiments and studies [20]. For FCN and mCL, the lowest tested concentration of 500 pmol L^−1^ could easily be detected. Only the signal heights for RuHex liposomes were lower than expected, resulting in a theoretical LOD slightly above 500 pmol L^−1^. RuHex and FCN usually resulted in similar signal heights, when using the same concentration or total lipid concentration. However, in the hybridization assay, the RuHex signals were only around 25% of the FCN signals at the same concentration. This fact also remained when using a different set of probes and liposome batches (Fig. S5). Additional experiments investigating possibly negative influences of free RuHex binding to DNA molecules [28] showed no effect (Fig. S6). Thus, it is assumed that the larger diameter of RuHex liposomes may result in fewer binding events on the magnetic beads. Hence, an increase in reporter probe concentrations was studied (see below).

Surprisingly, in a multiplex assay, in which all three liposomes but only one specific target sequence was present, significant non-specific binding was observed (Fig. S7). Here, not only the signals from the matching liposome were found, but also from the other two liposomes. Signal heights and signal ratios were also similar regardless which target present was present. This was independent of buffer composition, liposome combinations, incubation time and liposome concentration. (Fig. S7, S9, S10). Also no DNA cross-hybridization (Fig. S8), liposome fusion or aggregation (Fig. S11) was identified using respective experiments and in addition some DLS and NTA studies. We assume an exchange of reporter probes between liposomes, which has been reported several times in literature in other instances [29–32]. This could either be a result of the relatively weak single-cholesterol anchor, which spontaneously incorporates into lipid bilayers, therefore allowing transfer from one liposome to another [29–31], or lipid mixing as a precursor of liposome fusion [32]. DNA tagging can be utilized to promote liposome fusion by bringing liposomes with complementary sequences into close proximity. The amount of fusion depends on many parameters like lipid composition, type of DNA anchor, number of DNA on the surface, linker length between liposome and DNA, and the complementarity of the used DNA sequences [29, 30, 32]. While fusion and content mixing are much rarer and require precise parameter control, the much more prominent lipid mixing was also observed for liposomes with non-complementary sequences and even untagged liposomes [30–32]. This exchange of DNA can be overcome by two methods. Either stronger anchors like bivalent cholesterol anchors are used, which show an order of magnitude lower *k*_off_ value than single-cholesterol anchors, and therefore, irreversible coupling or sequential incubations can be used [30]. The latter was used in this study, but bivalent cholesterol anchors will be part of a future study.

In sequential incubation, a step with liposome 1 was followed by the addition of liposome 2 and then liposome 3. This results in non-specific binding of −0.4% to 25.1% (Fig. S12a). Interestingly, it was observed that the amount of non-specific binding depended on the respective liposome order, i.e. liposomes bound significantly less non-specifically, when they were incubated with the target before the correct liposome was added (−0.4% to 7.3% compared to 4.3% to 25.1%). This was expected, as the matching liposome is required for the probe exchange. Subsequent liposomes can then obtain matching reporter probes from liposomes bound on the MBs.

**Fig. 4:**
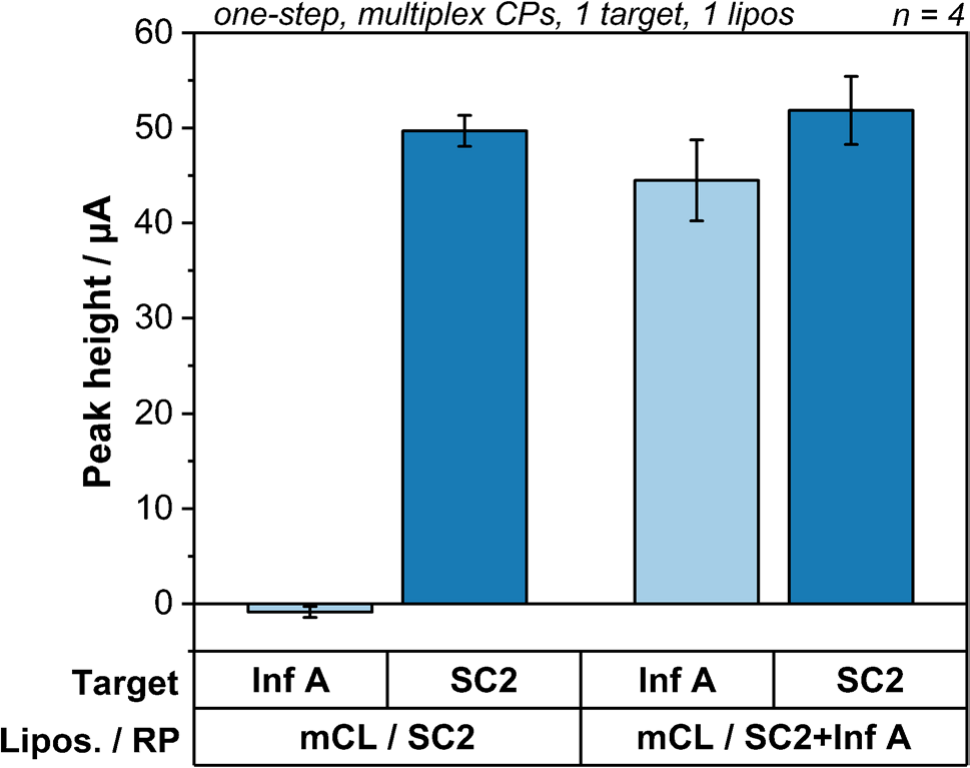
Binding study of double reporter probe-modified liposomes. Peak heights of singleplex assays with 25 nmol L^−1^ Inf A or SC2 targets using basic mCL/SC2 liposomes or mCL/SC2 liposomes modified with additional Inf A reporter probe

The spontaneous insertion of cholesterol was confirmed and exploited by adding free cholesterol-tagged reporter probe to the assay. Specifically, a second cholesterol-modified reporter probe was incubated with liposomes for 30 min before using them in the actual assays (Fig. 4). These double modified liposomes where then able to bind to the second target with a similar performance as to their original target.

This latter post-synthesis modification was used to study higher reporter probe concentrations on modified liposomes. As expected, considering that the reporter probe concentrations were optimized previously [33], no further signal improvements were obtained for FCN and mCL liposomes, whereas for the RuHex liposomes, an increase of 32% in signal height was determined, when doubling the amount of reporter probe (Fig. S13). As expected, liposomes modified with 10 or 100 times lower amounts of a second reporter probe led to strong signal decreases (Fig. S13b). Also, lower concentrations of total lipid and thus liposomes, but the same number of reporter probes, led to a strong signal decrease, when the total lipid concentration was decreased below 250 µmol L^−1^ total lipid (Fig. S13c). Thus, for the final assays, RuHex liposomes were synthesized ab origine with double the original reporter probe concentration, i.e. 0.026 mol%.

Finally, it was found that the presence of multiple capture probes on each MB leads to a competition for binding sites in scenarios with multiple targets present, which significantly favoured the first liposome, and leads to a decrease in signals for subsequent liposomes. By simply modifying a MB with only one capture probe and using thus three types of MB per assay, no more competition for binding sites occurred (Fig. S12). The loss in maximum signals height by having a lower number of potential binding sites per target was counteracted for the final dose-response curves by tripling the amount of magnetic beads used.

### Optimized multiplex assay for the simultaneous detection of all three analytes

**Fig. 5:**
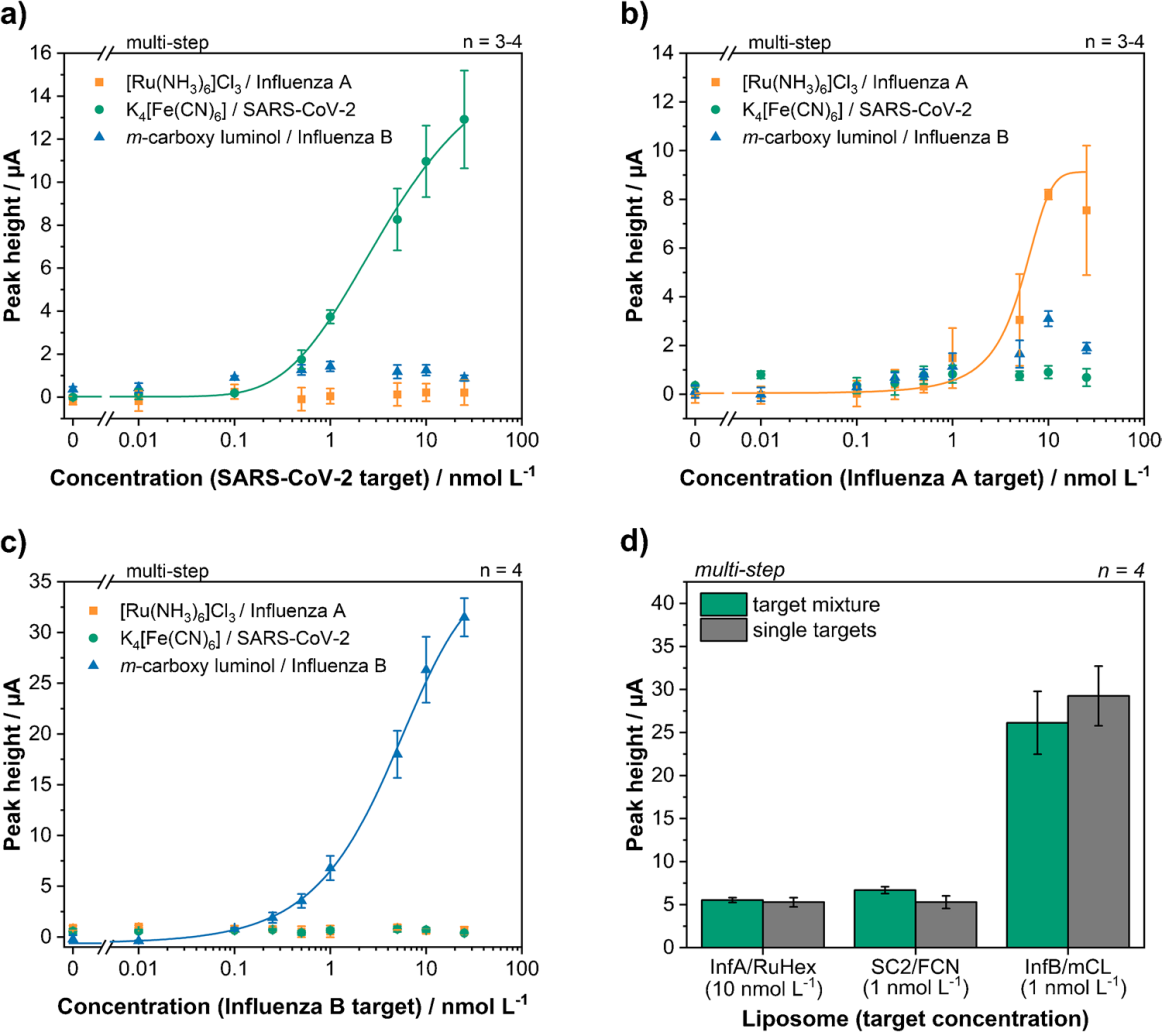
Multiplex dose-response curves for SC2 target (**a**), Inf A target (**b**) and Inf B target (**c**) in a concentration range between 0 and 25 nmol L^−1^. The matching liposome was always used first to simulate the worst-case scenario with the most amount of non-specific binding. (**d**) Comparison between a multiplex assay with all three targets present and the sum of three individual, multiplex assays with just one target present. Target concentrations and liposome incubation order were kept the same

Dose-response curves for each target were recorded in the sequential, multiplex assay. To simulate the worst-case scenario, i.e. most non-specific binding, a liposome order was chosen using the matching liposome always first (Fig. 5a–c). Limits of detections determined were 125 pmol L^−1^, 130 pmol L^−1^ and 1.6 nmol L^−1^ for Influenza B (mCL), SARS-CoV-2 (FCN) and Influenza A (RuHex), respectively. The LODs for mCL and FCN were only slightly worse than those obtained in a previous singleplex study using FCN liposomes with *C. parvum* DNA (47 pmol L^−1^) [20]. Such a slight increase in LOD is to be expected for multiplex assays [34]. The higher LOD for RuHex was caused by high standard deviations due to variations of the non-linear background signals in this potential region on LIG electrodes and the low signal intensities obtained during all DNA assays as indicated earlier. Different encapsulants should be investigated for DNA-based assays in the future, e.g. ferroin. The amount of non-specific binding in general increased with increasing target concentration as also more matching liposomes are bound on the magnetic beads and therefore present during the subsequent liposome incubations.

Viral loads for influenza and SARS-CoV-2 usually range from a few hundred copies per mL to 10^11^ copies per mL with means around 10^4^–10^6^ copies per mL [35–37]. Our lowest LOD of 125 pmol L^−1^ would be equivalent to 7.5∙10^10^ copies per mL. So very extreme cases might be detected, but for the majority of samples our sensitivity is several orders of magnitude too low and therefore amplification like NASBA would be required for clinical samples.

In Table 3, this work is compared to other related publications. It can be concluded that in general electrochemical detection cannot compete with fluorescence- or ToF-SIMS-based detection, when it comes to limits of detection, but the much simpler and less expensive instrumentations still make it the superior choice for point-of-care testing. The outperformance by other electrochemical approaches can be attributed to different assay principles, detection methods, a lower degree of multiplexing and/or different electrode materials and not necessarily because of the chosen liposomes and encapsulants. Most other studies rely on screen-printed electrodes with additional complex modifications. While this gives them better performances, the extremely simple, scalable and inexpensive fabrication of LIG [22] has to be considered for commercial applications.

**Table 3:** Comparison with other related work using liposomes or nanoparticles as labels for biosensing

Analyte	Encapsulant	Detection method	Assay type	LOD	Ref.
Inf B SARS-CoV-2 Inf A	mCL FCN RuHex	SWV	DNA hybridization	125 pmol L^−1^ 130 pmol L^−1^ 1600 pmol L^−1^	This work
ProGRP NSE	AA UA	LSV	Immunoassay	10 pg mL^−1^ 180 pg mL^−1^	[3]
Zearalenone Aflatoxin B1	Green QDs Orange QDs	Luminescence	Immunoassay	0.02 ng L^−1^ 0.01 ng L^−1^	[10]
HIV-1 HIV-2	Green QDs Red QDs	Fluorescence	DNA hybridization	Attomolar	[11]
*C. parvum*	FCN	SWV	DNA hybridization	47 pmol L^−1^	[20]
*E. coli* O157:H7 *Salmonella *spp. *L. monocytogenes*	SRB	Fluorescence	Array-based immunosorbent assay	3.1×10^3^ CFU mL^−1^ 7.8×10^4^ CFU mL^−1^ 7.9×10^5^ CFU mL^−1^	[38]
DNA	Cy5 Rhod-PE	Fluorescence	DNA hybridization	n.a.	[12]
Inf A H1N1 Inf A H3N2 Inf A H5N1 Inf B	Methylene blue Ru(bpy)_3_^2+^ Acridine orange Ferrocenium tetrafluoroborate (in SiNP)	DPV	DNA hybridization	1.6 pmol L^−1^ 1.6 pmol L^−1^ 4.7 pmol L^−1^ 2.9 pmol L^−1^	[34]
miRNA-155 miRNA-21 miRNA-16	PHSGNPs-Cd^2+^ PHSGNPs-Pb^2+^ PHSGNPs-Cu^2+^	DPV	DNA/RNA hybridization	0.98 fmol L^−1^ 3.58 fmol L^−1^ 0.25 fmol L^−1^	[25]
Aβ/Tau Biotin/GM1	-	Fluorescence and ToF-SIMS	Immunoassay	Single liposome	[8]
Oligonucleotides	-	ToF-SIMS	DNA hybridization	Single molecule	[9]
EGFR VEGF	Cd^2+^ Cu^2+^	PSA	Immunoassay	0.01 pg mL^−1^ 0.005 pg mL^−1^	[13]

Finally, exemplary studies were done to investigate effects of multiple DNA targets being present in one sample. Specifically, it was tested whether combinations of experiments with only one target can be used to predict and therefore calibrate future multiplex experiments. Here, the results of exemplary mixtures containing all three targets were compared to the sum of three individual assays (Fig. 5d, S14). Target concentration and liposome order were kept the same. A good correlation of 89–126% was found between the sum of individual assays and the multiplex assay. Therefore, this will be used for calibration and evaluation in the future including a simple mathematical model for data interpretation. Also, good reproducibility was found throughout different multiplex assays with average relative standard deviations of 15.2%, 8.3% and 7.0% for RuHex, FCN and mCL respectively.

## Conclusion

Electrochemistry is of particular interest for multiplexing strategies due to the large variety of redox markers with different redox potentials available and due to its simplicity of use for quantitative point-of-care applications. Here, an electrochemical liposome-based multiplex platform was developed using nucleic acid sequences as model analytes. RuHex, FCN and mCL were identified as multiplex redox markers for simultaneous electrochemical detection and successfully encapsulated into DNA-modified liposomes with excellent long-term stability over the course of at least 12 months. These liposomes were applied to the multiplex detection of NASBA amplicons derived from Influenza A, Influenza B and SARS-CoV-2 with limits of detections of 1.6 nmol L^−1^, 125 pmol L^−1^ and 130 pmol L^−1^, respectively. The unexpected underperformance of RuHex liposomes could be explained partly by the increased liposome size but requires further studies in the future. It would be of particular interest to determine, if similar effects are observed for other biorecognition elements. Otherwise ferroin might be a potential replacement in the current marker selection for a triplex system.

Furthering knowledge of previous studies [29, 39], it was found that liposomes could be easily modified post synthesis with sufficient cholesterol-tagged reporter probes through simple incubation reactions. However, this also led to undesired binding in multiplex settings caused by the exchange of reporter probes between liposomes and needed to be overcome by separating the incubation steps of liposomes. Future studies should investigate whether this is only related to the single-cholesterol anchor and could be prevented by different anchoring strategies and if this is only relevant for DNA-based assays or also other biorecognition strategies. These findings could then allow the realization of a desired one-step multiplex assay and subsequent integration into a microfluidic system for on-chip multiplex NASBA amplification and multiplex detection similar to previous singleplex systems [40]. While the additional requirements for marker encapsulation into liposomes make it currently more challenging to increase multiplexing capabilities compared to conventional labels, the multiplexing capabilities of electrochemical liposomes were increased in this work from the typical duplex detections [3, 13] to a triplex detection. Different liposome preparation methods might allow the use of a larger variety of redox markers including hydrophobic ones and open up new and additional encapsulant combinations with superior performances over labels without intrinsic amplification. Encapsulants worth investigation in the future are for example anthraquinone, thionine, ferrocene carboxylic acid, Co(bpy)_3_^3+^ [24], Cd^2+^, Cu^2+^, Zn^2+^ and Pb^2+^ [13, 25, 26].

### Supplementary Information

Below is the link to the electronic supplementary material.

Supplementary file1 (PDF 1.03 MB)
