# Supplementary material for: Multiplexed electrochemical liposomes applied to the detection of nucleic acids for Influenza A, Influenza B and SARS-CoV-2
Source: Anal Bioanal Chem. 2024 Jan 19;416(15):3487–500. doi: 10.1007/s00216-024-05145-8 (PMC11156727; doi:10.1007/s00216-024-05145-8)
Supplement: Supplementary file 1 — Supplementary file1 (PDF 1.03 MB) [file 216_2024_5145_MOESM1_ESM.pdf]

Analytical and Bioanalytical Chemistry

Supporting Information

Multiplexed Electrochemical Liposomes applied to the Detection of Nucleic Acids for Influenza A, Influenza B and SARS-CoV-2

Florian Gerstl, Michael Loessl, Veronika Borggraefe, Antje J. Baeumner

Institute of Analytical Chemistry, Chemo- and Biosensors, University of Regensburg, Universitätsstraße 31, 93053 Regensburg, Germany

*Corresponding author: Antje J. Baeumner, antje.baeumner@ur.de

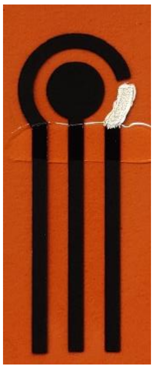

Fig. S1 *LIG electrode used for all electrochemical measurements. It consits of LIG working and counter electrode and a pseudo Ag/AgCl reference electrode made with silver ink. The electrode area is restricted by nail polish.*

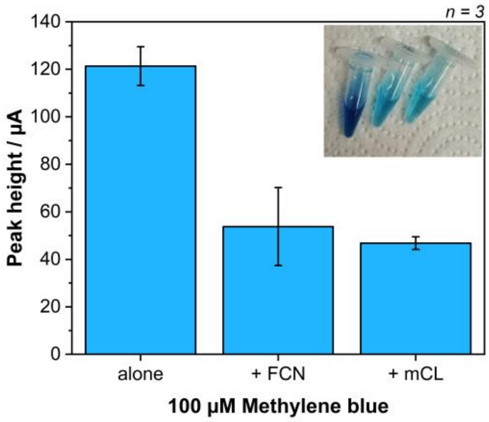

Fig. S2 *Cross-reactivity study of methylene blue with FCN and mCL. Peak heights of 100 µmol L^{-1} methylene blue alone, in mixture with FCN and in mixture with mCL. The insert shows images the respective solution. The blue color decreases, when methylene blue gets reduced by FCN or mCL.*

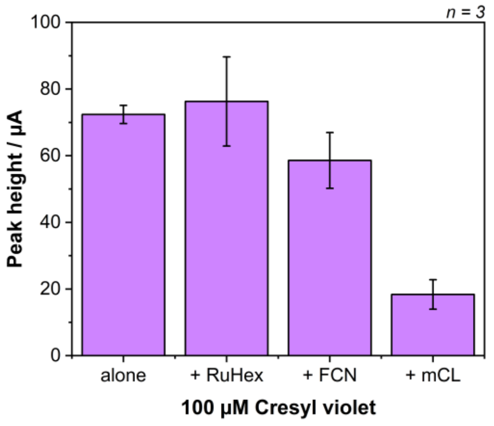

Fig. S3 *Cross-reactivity study of 100 µmol L⁻¹ cresyl violet in mixture with with 100 µmol L⁻¹ RuHex, FCN or mCL.*

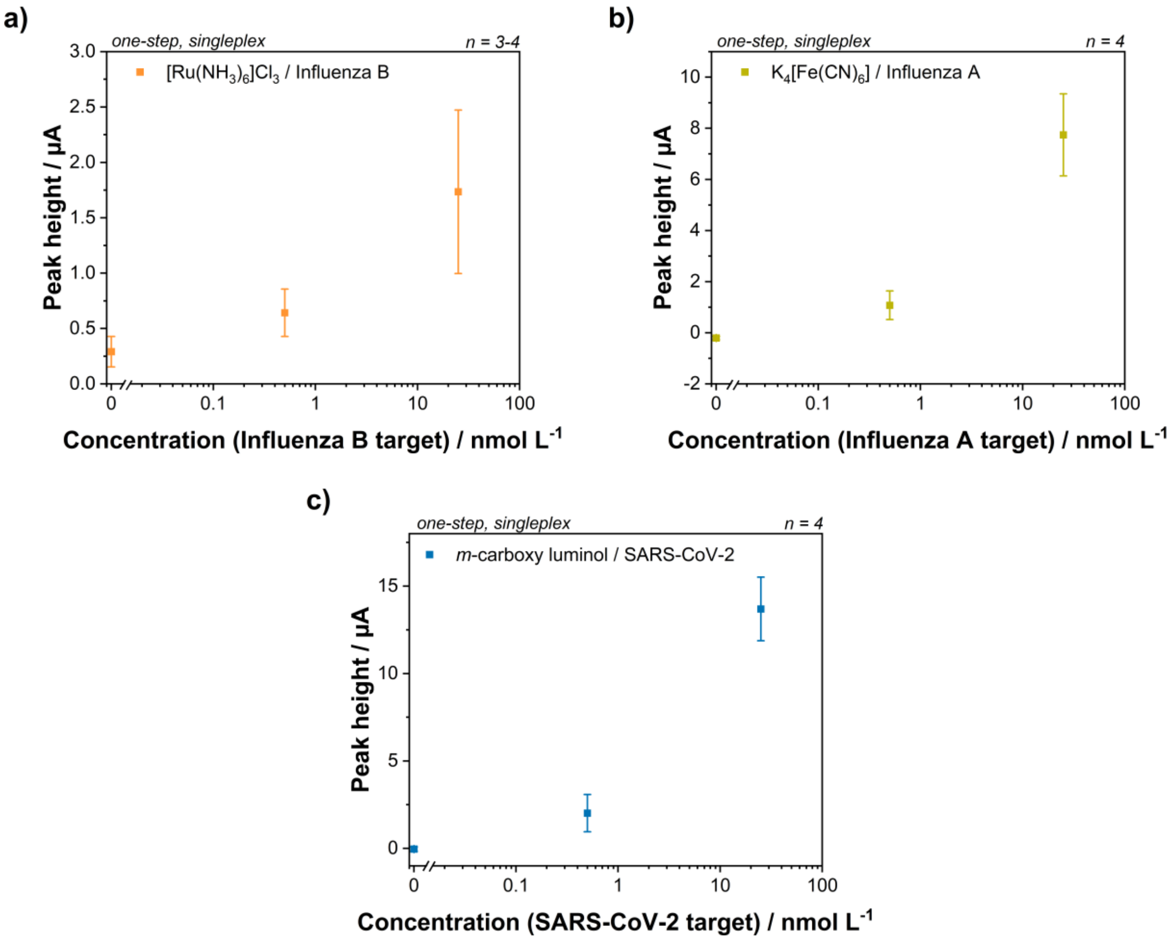

Fig. S4 *Hybridization assay test with new liposomes. Peak heights obtained from one-step, singleplex assays using only one liposome and its respective target DNA at concentrations of 0, 0.5, 25 nmol L⁻¹ for a) InfB target and InfB/RuHex liposomes, b) Inf A target and Inf A/FCN liposomes and c) SC2 target and SC2/mCL liposomes.*

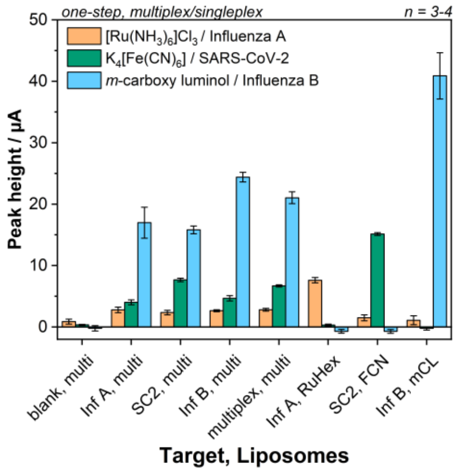

Fig. S5 *Hybridization assays with liposomes with new encapsulant-reporter probe combinations. Liposomes were tested in one-step, multiplex assays with either one or all three targets present. And in singleplex assays with just their matching target present to confirm binding functionality. Tagret concentrations used were always 25 nmol L*$^{-1}$.

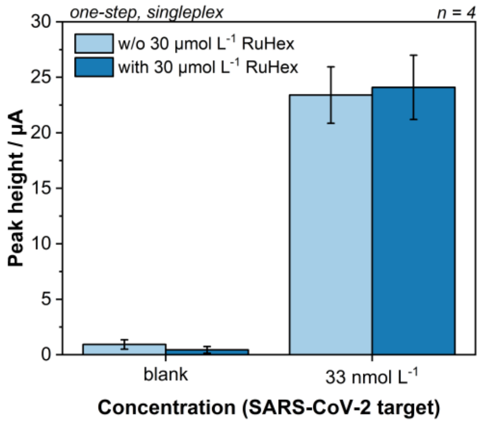

Fig. S6 *Influence of free RuHex on DNA hybridization. One-step, singleplex assays with mCL/SC2 liposomes and SC2 target (0 and 33 nmol L*$^{-1}$*) with/without additional 30 µmol L*$^{-1}$ *RuHex in the hybridization buffer.*

Multiplex buffers

The buffer composition was investigated, as it has a large influence on DNA hybridization. With increasing stringency (e.g. lower salt concentration, higher formamide content) it becomes more difficult for mismatched DNA strands to hybridize until only perfectly matching sequences can hybridize. However, no effect on the non-specific signals was observed, when changing the composition of the hybridization buffer or washing buffer (Fig. SI7a). The stringency of the hybridization buffer was increased by decreasing the SSC concentration from 9× to 6× or 3×. However, only a general decrease in hybridization for all DNAs was observed, with no influence on the non-specific signals. When increasing the stringency even further for the washing buffer by changing to 1xSSC and increasing the formamide content, again just an overall decrease of signals was observed, but no effect on the non-specific signals (Fig. SI7b). Other buffers like PBS or Tris-HCl also showed no improvements. These results were surprising at first, as decreasing signals from the liposomes with non-matching probes had been expected. This can only be explained by the fact, that all obtained signals result from correct hybridizations, which heavily supports the theory of reporter probe exchange between liposomes[29-32]. In that case, matching reporter probes are carried by all liposomes including those intended as labels for other targets.

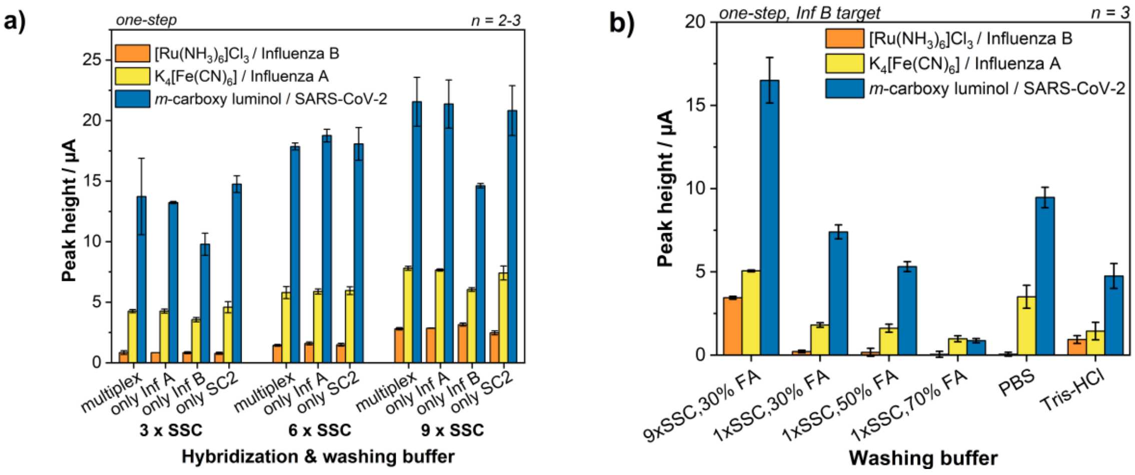

Fig. S7 *a) Influence of hybridization and washing buffer on one-step multiplex assays. Peak heights from assays with either Inf A, Inf B or SC2 target or all three targets present in different hybridization buffers with varying SSC content (3x,6x,9x). Targets were always used at 25 nmol L^{-1}. b) Influence of washing buffers on one-step multiplex assays. Peak heights from multiplex assays with only Inf B target using different washing buffers. The buffers tested were standard hybridization buffer, hybridization buffers with decreased SSC content and increased formamide (FA) concentration, PBS and Tris-HCl.*

Hybridization specificity between probes and target

The interactions between capture probe and target (Fig. SI8a) and between reporter probe and target (Fig. SI8b) were investigated in more detail in separate experiments to confirm probe specificity. In both experiments the results can be summarized by two scenarios. Either the assay mixture contains a complete set consisting of matching probes, target and liposomes, which results in signals from all present liposomes, including unintended signals. Or there are no signals, if no matching set is present. These results were also true, when using liposomes and probes against *Cryptosporidium parvum* from previous studies[20]. Therefore, unwanted cross-hybridization caused by insufficient probe design could be excluded to be responsible for the non-specific signals.

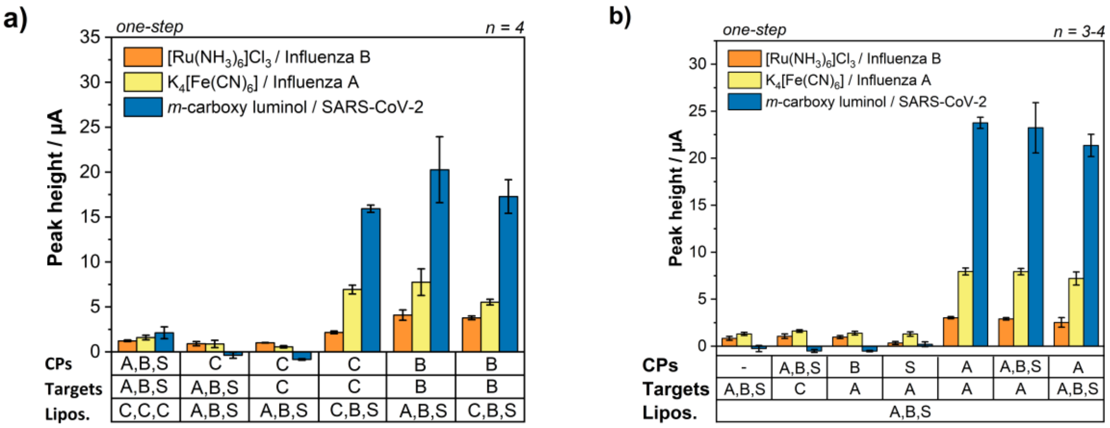

***Fig. S8** a) Reporter probe specificity study. One-step multiplex assays with matching capture probe and target but different reporter probe modified liposomes b) Capture probe specificity study. One-step multiplex assays with different capture probe and target combinations or no capture probe.*

One-step hybridization assays with two liposomes

Since RuHex liposomes showed consistently low signals and different behaviours in DLS measurements (data not shown), experiments with only two liposomes (one matching, one non-matching liposome) were carried out to investigate, whether just one liposome is causing problems resulting in the non-specific signals. Therefore, all possible combinations just two liposomes were tested (Fig. SI9). In all cases, signals from both the matching and the non-matching liposome were obtained. Thus, it was concluded, that the non-specific signals are not caused by one liposome alone.

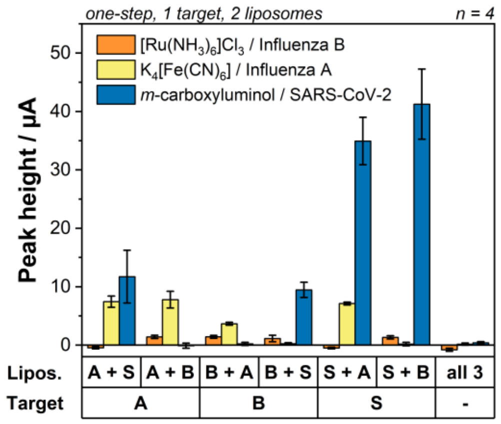

Fig. S9 *Assays with only two liposomes. One-step multiplex assays with one target, one matching liposome and one non-matching liposome. Target concentration was always 25 nmol* L^{-1}

Assay time and liposome concentration

Most scenarios that could explain the non-specific binding require interaction between liposomes. Therefore, incubation time and liposome concentration were investigated (Fig. SI10). An incubation time of 10 min, 30 min or 90 min had no effect on the signal height. This also demonstrates a fast assay time. Decreasing the liposome concentration from 500 µmol L^{-1} to 50 µmol L^{-1} only led to the expected decrease in overall signals but had no effect on the non-specific signals.

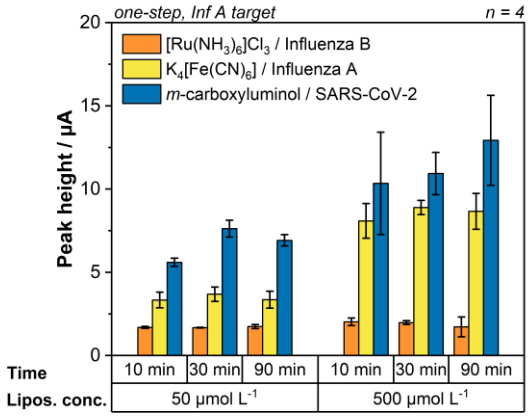

Fig. S10 *Influence of incubation time and liposome concentration on multiplex assays. One-step multiplex assays with only Inf A target using different incubation times (10 min, 30 min, 90 min) and liposome concentrations (50 µmol L^{-1}, 500 µmol L^{-1}).*

Liposome fusion or aggregation

A potential aggregation or fusion of liposomes, that would explain non-specific signals, was investigated by DLS measurement (Fig. SI12). Measurements of individual liposomes were compared to mixtures of liposomes. No increase in liposome size between mixture and single measurements was observed, hinting at fusion or aggregation. In hybridization buffer all liposome peaks broadened, resulting in an increase in PdI (data not shown). It seems, that liposome morphology is affected in this buffer, but the non-specific signals also occurred in other buffers like HSS buffer, where this broadening effect was not observed.

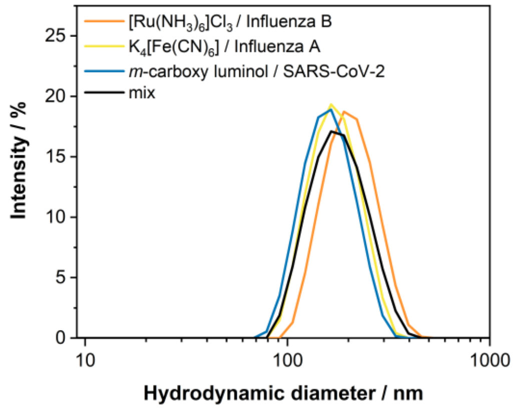

Fig. S11 *Size by intensity determined through DLS measurements in HSS buffer of RuHex/Inf B, FCN/Inf A and mCL/SC2 liposomes and a mixture of all three liposomes.*

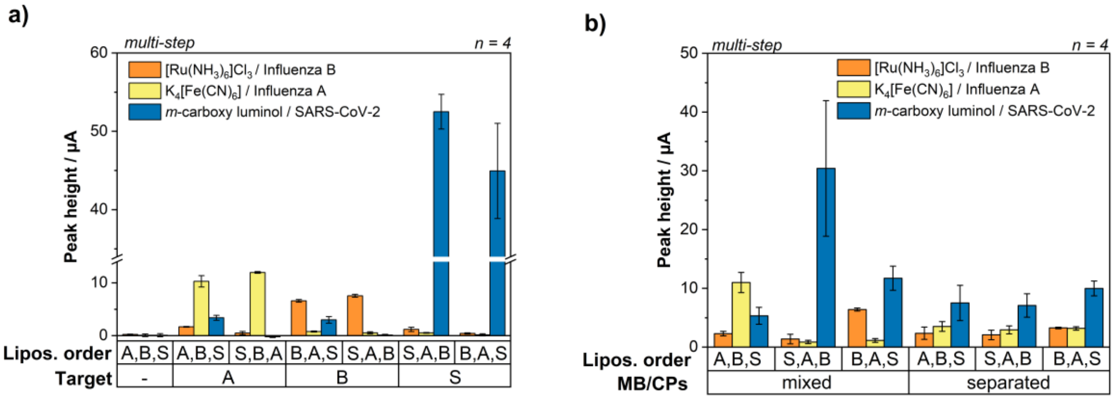

Fig. S12 *a) Multistep assays with separated liposome incubations in different orders. For every target the matching liposome was once incubated first and once incubated last. Target concentration was always 25 nmol L^{-1}. b) Influence of liposome incubation order in combination with MB mixed CPs or separated MB for every capture probe. Stepwise multiplex assays with all targets present at 25 nmol L^{-1} but different liposome incubation orders. Each liposome was used first once. Same orders were tested once with mixed MB/CPs and once with separated MB/CPs.*

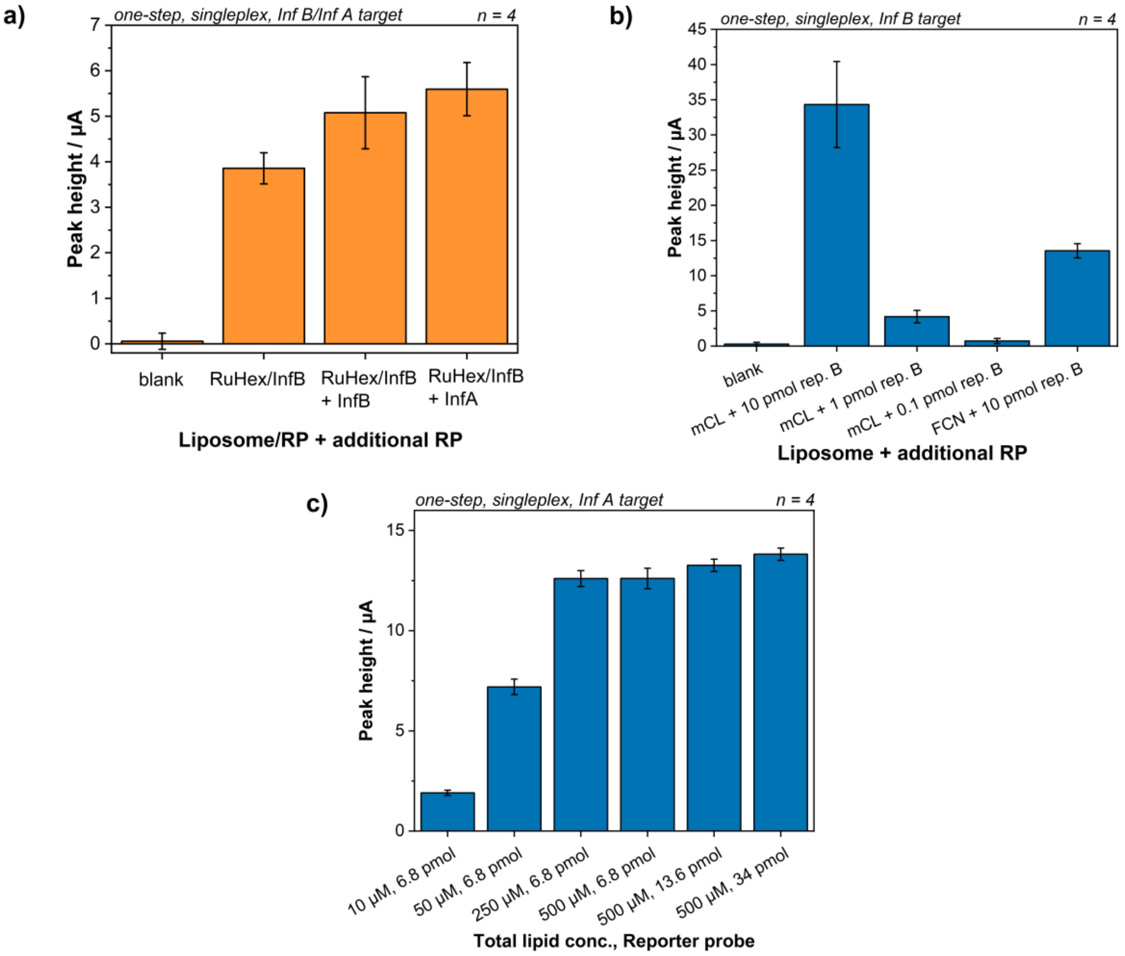

Fig. S13 *Studies on post-synthesis dual-modified liposomes. Target concentrations were always 25 nmol L-1. a) Peak heights from hybridization assays with RuHex/Inf B liposomes, RuHex/Inf B liposomes modified with more Inf B reporter probe using Inf B target and RuHex/Inf B liposomes modified with Inf A reporter probe tested against Inf A target. b) mCL/SC2 liposomes modified with varying amounts of additional Inf B reporter probe (0.1, 1, 10 pmol) and tested against Inf B target. c)* FCN/Inf A *liposomes with different ratios between total lipid concentration (10-500µmol L-1) and amount of Inf A reporter probe (6.8 – 34 pmol) tested against* Inf A *target.*

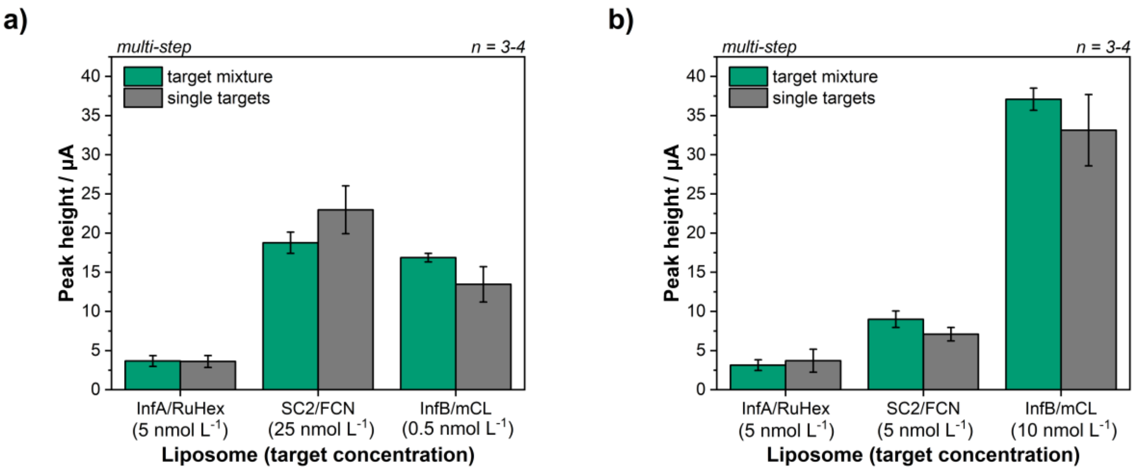

Fig. S14 *Comparison between multiplex assays with all three targets present and the sum of three individual, multiplex assays with just one target present. Target concentrations and liposome incubation order were kept the same.*

References

[20] Gerstl F, Pongkitdachoti U, Unob U, Baeumner AJ. Miniaturized sensor for electroanalytical and electrochemiluminescent detection of pathogens enabled through laser-induced graphene electrodes embedded in microfluidic channels. Lab Chip. 2022;22(19):3721-3733. https://doi.org/10.1039/D2LC00593J

[29]; Pfeiffer I, Höök F. Bivalent Cholesterol-Based Coupling of Oligonucletides to Lipid Membrane Assemblies. J Am Chem Soc. 2004;126(33):10224-10225. https://doi.org/10.1021/ja048514b

[30] Stengel G, Zahn R, Höök F.DNA-Induced Programmable Fusion of Phospholipid Vesicles. J Am Chem Soc. 2007;129(31):9584-9585. https://doi.org/10.1021/ja073200k

[31] Chan YHM; van Lengerich B; Boxer SG. Lipid-anchored DNA mediates vesicle fusion as observed by lipid and content mixing. Biointerfaces. 2008;3(2):FA17-FA21. https://doi.org/10.1116/1.2889062

[32] Chan YHM, van Lengerich B, Boxer SG. Effects of linker sequences on vesicle fusion mediated by lipid-anchored DNA oligonucleotides. Biol sci. 2009;106(4):979-984. https://doi.org/10.1073/pnas.0812356106
